# The impact of native Fallot anatomy on future therapeutic requirements and outcomes at follow-up

**DOI:** 10.1186/s12947-021-00249-y

**Published:** 2021-06-19

**Authors:** Antonio Ravaglioli, Lamia Ait-Ali, Duccio Federici, Stefano Salvadori, Arketa Pllumi, Vitali Pak, Chiara Marrone, Alessandra Pizzuto, Philipp Bonhoeffer, Pierluigi Festa

**Affiliations:** 1 Division of Pediatric Cardiac Surgery, Fondazione G. Monasterio, Massa, Italy; 2 Institute of Clinical Physiology, National Research Council (CNR), Via Aurelia Sud, 54100 Massa, Italy; 3 Faculty of Natural Sciences, Shkodër, Albania; 4 Division of Pediatric Cardiology, Fondazione G. Monasterio, Massa, Italy; 5 FTGM, Massa, Italy

**Keywords:** Tetralogy of Fallot, Native anatomy, Surgical/interventional procedure, Follow-up

## Abstract

**Background:**

In patients with repaired Fallot, subsequent surgical or interventional procedures and adverse cardiac events are frequent. We aimed to evaluate the impact of a simple pre-operative anatomic classification based on the size of the pulmonary valve (PV) annulus and branches on future therapeutic requirements and outcomes.

**Method:**

This is a single-center retrospective analysis of patients operated for Fallot before the age of 2 years, from January 1990. Pre-operative anatomy, surgical and interventional procedures and adverse events were extrapolated from clinical records.

**Results:**

Among the 312 patients, a description of the PV and pulmonary arteries (PAs) native anatomy was known in 239 patients (male:147, 61.5%), which were divided in the following 3 groups: group 1 (65 patients) with normal size of both PV and PAs; group 2 (108 patients) with PV hypoplasia but normal size PAs; group 3 (66 patients) with concomitant hypoplasia of the PV and PAs. During the 12.7 years (IQR 6.7–17) follow-up time, 23% of patients required at least one surgical or interventional procedure. At Kaplan–Meier analysis, there was a significant difference in requirement of future surgical or interventional procedures among the 3 groups (*p* < 0,001). At multivariate Cox regression analysis, hypoplasia of PV and PAs was an independent predictor of subsequent procedures (HR:3.1,CI:1.06–9.1, *p* = 0.03).

**Conclusion:**

Native anatomy in Tetralogy of Fallot patients affects surgical strategy and follow-up. It would be therefore advisable to tailor patient’s counseling and follow-up according to native anatomy, rather than following a standardized protocol.

**Supplementary Information:**

The online version contains supplementary material available at 10.1186/s12947-021-00249-y.

## Background

La Maladie Bleue, as described by Louis Arthur Etienne Fallot, depicts the physiology created by a combination of anatomic malformations, known as Tetralogy of Fallot (TOF) [1, 2]. Its pivotal features include a typical ventricular septal defect (VSD) associated to an anterior displacement of the infundibular septum, leading to different degrees of overriding aorta and obstruction of the right ventricular outflow tract (RVOT) at several levels [3]. Nevertheless, TOF includes a wide anatomical and clinical spectrum, mainly determined by the morphology of the RVOT, and by the size of the pulmonary valve (PV) and of the pulmonary artery branches (PAs). The original operation for TOF repair was performed through a large ventriculotomy, often using a transannular RV patch [4, 5]. Several studies however demonstrated the negative consequences of these surgical techniques, as causing pulmonary regurgitation, right ventricular (RV) enlargement and dysfunction, reduced exercise capacity, arrhythmias and heart failure (HF) [6–8]. In recent times, many efforts have been made to minimize the extent of the ventriculotomy and to preserve the PV function. Nonetheless, the size of the native PV still remains the main limiting factor.

In this study, we aimed to evaluate the impact of a simple pre-operative anatomic classification based on the size of the pulmonary valve annulus and pulmonary artery branches on future therapeutic requirements and outcomes.

## Materials and methods

We retrospectively identified all patients under the age of 2 years who underwent isolated TOF repair in our hospital between 1990 and 2019. Those with TOF and associated other defects such as absent pulmonary valve, pulmonary atresia, major aorto-pulmonary collaterals, atrio-ventricular septal defect and Ebstein-like tricuspid valve were excluded from the study. The study was approved by the Institutional Review Board of our hospital.

Medical history, pre-operative transthoracic echocardiogram (TTE) and operative reports of each patient were thoroughly reviewed. Based on the TTE results, the PV and PAs were considered hypoplastic if the z-score was less than -2. Whenever preoperative TTE was not available, we estimated the values based on the size of the Hegar dilator used during surgery, stated in the operative reports. Therefore, according to the size of the PV annulus and PAs, the study population was divided in 3 groups: group 1 included patients with normal PV and PAs size; group 2 patients with PV hypoplasia but normal PAs size and group 3 those with both PV and PAs hypoplasia.

Follow-up data were obtained from hospital records, out-patient clinic letters or by telephone interview. All percutaneous and surgical procedures that were performed after complete repair were recorded, including those done in other hospitals. The cardiac events that occurred during the follow-up were also considered. Those included aborted sudden death and all cardiac deaths, sustained and non-sustained ventricular tachycardia (VT/NSVT), sustained supra-ventricular tachycardia (ectopic atrial tachycardia, atrial flutter or atrial fibrillation) and implantation of pacemaker or implantable cardiac defibrillator (ICD).

### Surgical interventions

Before definitive TOF repair, an initial modified Blalock-Taussig (BT) shunt was performed to palliate patients with of inadequate pulmonary circulation less than 3 months old or < 3.5 kg of body weight. Those were defined as being prostaglandin dependent or having low systemic arterial saturations (< 80%) or frequent cyanotic spells. The procedure, which was performed through median sternotomy without cardiopulmonary bypass support, entailed a shunt from the brachiocephalic artery to the pulmonary artery using a 3 mm or 3.5 mm polytetrafluoroethylene (PTFE) tube (W.L. Gore & Associates (UK) Ltd).

The TOF repair procedure was instead performed on cardiopulmonary bypass with bi-caval cannulation and moderate hypothermia. The RVOT muscle resection and the VSD closure were typically performed through the right atrium. If the PV was stenotic or dysplastic, then a commissurotomy was also carried out. In case of hypoplastic PV, a transannular patch (TAP) of glutaraldehyde-treated autologous pericardium or bovine pericardium was used. An infundibular patch was fashioned in patients with adequate PV annulus and small RVOT, despite an extensive muscle resection. An autologous or bovine pericardium patch was used also in those patients who required enlargement of the main pulmonary trunk or the PAs. Whenever present, the BT shunt was closed and divided at the time of the complete repair. At the end of the procedure, after the modified ultrafiltration, the RV/LV pressure ratio was considered acceptable if < 0.75.

### Data analysis

Continuous variables are expressed as mean ± standard deviations or median with IQR 25–75%. Dichotomous variables are expressed as absolute numbers and percentages. A *p* value of < 0.05 was considered statistically significant. The comparison between groups for continuous variables was performed by analysis of variance (ANOVA). Unadjusted associations were assessed using Chi-square test. In order to establish the effect of a single covariate on subsequent procedures, univariate Cox regression models were built. The covariates found to be significant at the univariate analysis (*p* < 0.05) were included in the multivariate analysis. Kaplan–Meier methods with the log-rank test and Cox proportional hazards regression analysis were used to analyze the freedom from re-operation or re-interventional procedure at follow-up.

## Results

### Population study

Between January 1990 and December 2019, 312 patients aged less than 2 years with TOF and DORV-Fallot type, underwent complete surgical repair. In 239 patients (male: 147, 61.5%, 8.2 ± 3.7 patients/year), a description of the PV and PAs native anatomy was available, thus they were considered for the present study. Among them, 16.3% were initially palliated as previously described, mainly with modified BTS. Only 2 patients underwent a different palliative operation, RVOT and PDA stenting, both performed in 2018. The median age at the time of complete repair was 0.67 years (IQR 0.51–0.9). The infundibular resection was performed routinely in all patients to release the subvalvular stenosis. A TAP was used in 164 patients (68.6%), while in 75 patients the PV was preserved: an infundibular patch in 39 patients (16.3%) and a combined RVOT muscle resection and PV commissurotomy in 46 patients (19.2%). The main PA trunk was enlarged with pericardial patch in 10 patients with preserved PV to release the supravalvular stenosis.

There were 8 early deaths (3.3%).

### Surgical repair according to the native RVOT and PAs anatomy

Among the 239 patients, 65 (27.2%) were in group 1; 108 (45.2%) in group 2; 66 (27.6%) in group 3. Demographic data and surgical history are summarized in Table 1.

**Table 1:** Surgical repair related to pre-surgical anatomy

	**Group 1 (65)**	**Group 2 (108)**	**Group 3 (66)**	***P***
**Gender male n (%)**	50 (77)	58 (54)	39 (59)	**0.009**
**Previous BT shunt n (%)**	8 (12)	8 (7.4)	21 (31.8)	**0.001**
**PDA stent**		1		
**RVOT stent**		1		
**Age at complete repair (years)**	0.66 (0.48–0.92)	0.67 (0.55–0.92)	0.7 (0.52–1)	0.78
**Type of repair n (%)**				** < 0.0001**
**TAP**	4 (6.2)	98 (91)	62 (94)	
**With PAs enlargement**	0	2	44	
**Without PAs enlargement**	4	96	18	
**Infundibular patch**	26 (40)	10 (9.4)	3 (4.5)	
**With PAs enlargement**	1	0	2	
**Without PAs enlargement**	25	10	1	
**RVOT muscle resection ± PV commissurotomy**	35 (53.8)	8 (7.5)	3 (4.5)	

*PAs* Pulmonary artery branches, *PDA* Patent ductus arteriosus, *PV* Pulmonary valve, *RVOT* Right ventricular outflow tract, *TAP* Trans annular patch

Most of the patients in group 1 were male and only 6.2% of them underwent a TAP repair, and the PV was preserved in 93.8%. As expected, 91% of the patients in group 2 required instead TAP repair and in only 9% of this group the PV was preserved. In group 3, 31.8% of the patients were initially palliated with a BT shunt and a TAP was required in 94% of them whereas the pulmonary valve was preserved in 6% of this group. There was no difference in age, at the time of the primary repair, between the 3 groups.

### Follow-up according to the native RVOT and PAs anatomy

Among the 239 patients with known pre-operative anatomy, 61 patients (13, 23 and 25 patients from group 1, 2 and 3, respectively) were lost at follow-up, thus 178 patients were enrolled in the statistical analysis. There was no significant difference between patients lost at follow-up and the remaining population regarding gender, diagnosis, BT shunt palliation and type of complete repair; however, the age at the time of the repair was significantly higher in the patients lost at follow-up (0.77 (0.55;1.28) versus 0.66 (0.51;0.87), *p* = 0.03) (supplement table 1). The median follow-up time from primary repair was 10.1 years (IQR 4.5–15.1) and the median age at last follow-up was 13.3 years (IQR 7.3–17.8).

Only 4 patients (2.2%), 3 in group 2 and 1 in group 3, experienced adverse cardiac events during the follow-up period (freedom form cardiac event at 20 years of follow-up was 93.3%). In group 2 one patient had a symptomatic VT with subsequent ICD implant at 13 years of age; another patient with history of coronary artery injury during primary repair required heart transplant at 10 years of age; the third patient had a traumatic rupture of pacemaker wires. In group 3, the only patient who suffered from cardiac adverse event required hospital admission at age 26 for decompensated HF secondary to RV diastolic dysfunction.

After the Fallot repair, 41 patients (23%) required further surgical or interventional procedures. Those were in total 51, as 10 patients required more than one procedure (Table 2). The mean time from complete repair to the first procedure was 10.3 ± 6.7 years. The first procedure was surgical in 26 patients and interventional in 15. There was no difference in time between primary repair and subsequent surgical of interventional procedure among the 3 groups. During the first 5 years following complete repair, residual RVOT obstruction was the main indication for surgical re-intervention in group 1 (2 patients) and in group 2 (5 patients); moreover, 1 patient in group 2 underwent pulmonary angioplasty due to supra-valvular stenosis and PAs angioplasty 3.5 years post complete repair. Most of the patients in group 3 instead, underwent surgical or interventional procedures for PAs stenosis. A significant number required surgical or interventional procedures to readdress RVOT/PV problems. Eight patients (19.5%) required multiple procedures. The number of patients in group 3 who underwent multiple procedures was 8, and this was significantly higher compared to group 2 (2 patients) and group 1 (no multiple re-interventions) (Table 2).

**Table 2:** Post-operative procedures related to pre-surgical anatomy

	**Total (178)**	**Group 1 (52)**	**Group 2 (85)**	**Group 3 (41)**	***P***
**Adverse cardiac event, n (%)**	4 (2.2)	0	3 (3.5)	1 (2.5)	0.3
**Pt underwent postop. procedure, n (%)**	41 (23)	3 (5.8)	16 (19.5)	22 (55)	** < 0.0001**
**F-up from primary repair, years (IQR)**	10.1 (4.5–15.1)	11.4 (5–16.9)	9.5 (4.7–15)	9.9 (2.4–14.7)	0.3
**Type of first procedure, n (%)**					0.4
**-Surgical**	26 (63.4)	2 (66.7)	12 (75)	12 (54.5)	
**-Interventional**	15 (36.6)	1 (33.3)	4 (25)	10 (45.5)	
**Number of procedures per pt**	0.3 (0–4)	0.07 (0–1)	0.24 (0–2)	0.87(0–4)	** < 0.001**
**-1 (%)**	31 (18)	3 (5.8)	15 (18.5)	13 (32.5)	
**-2 (%)**	5 (2.9)	0	2 (2.5)	3 (7.5)	
**-3 (%)**	4 (2.3)	0	0	4 (10)	
**-4 (%)**	1 (0.6)	0	0	1 (2.5)	
**Interventional procedure, n (%)**	21(12.2)	1 (2)	5 (6.2)	15 (37.5)	** < 0.001**
**-Angioplasty/stent PAs**	14	1	2	11	
**-Stent RVOT**	3	0	2	1	
**-Others**	4	0	1	3	
**Surgical procedures, n (%)**	30 (17)	3 (5.5)	11 (13)	16 (40)	**0.006**
**-RVOT muscle resection/patch enlargement**	8	2	5	1	
**-PVR + RVOT + PAs patch enlargement**	3	0	0	3	
**-RVOT + PAs patch enlargement**	2	0	1	1	
**-PVR/ RV-PA conduit implant**	14	1	5	8	
**-PAs patch enlargement**	3	0	0	3	

*PAs* Pulmonary artery branches, *PVR* Pulmonary valve replacement, *RV-PA* Right ventricle to pulmonary artery, *RVOT* Right ventricular outflow tract

**Fig. 1:**
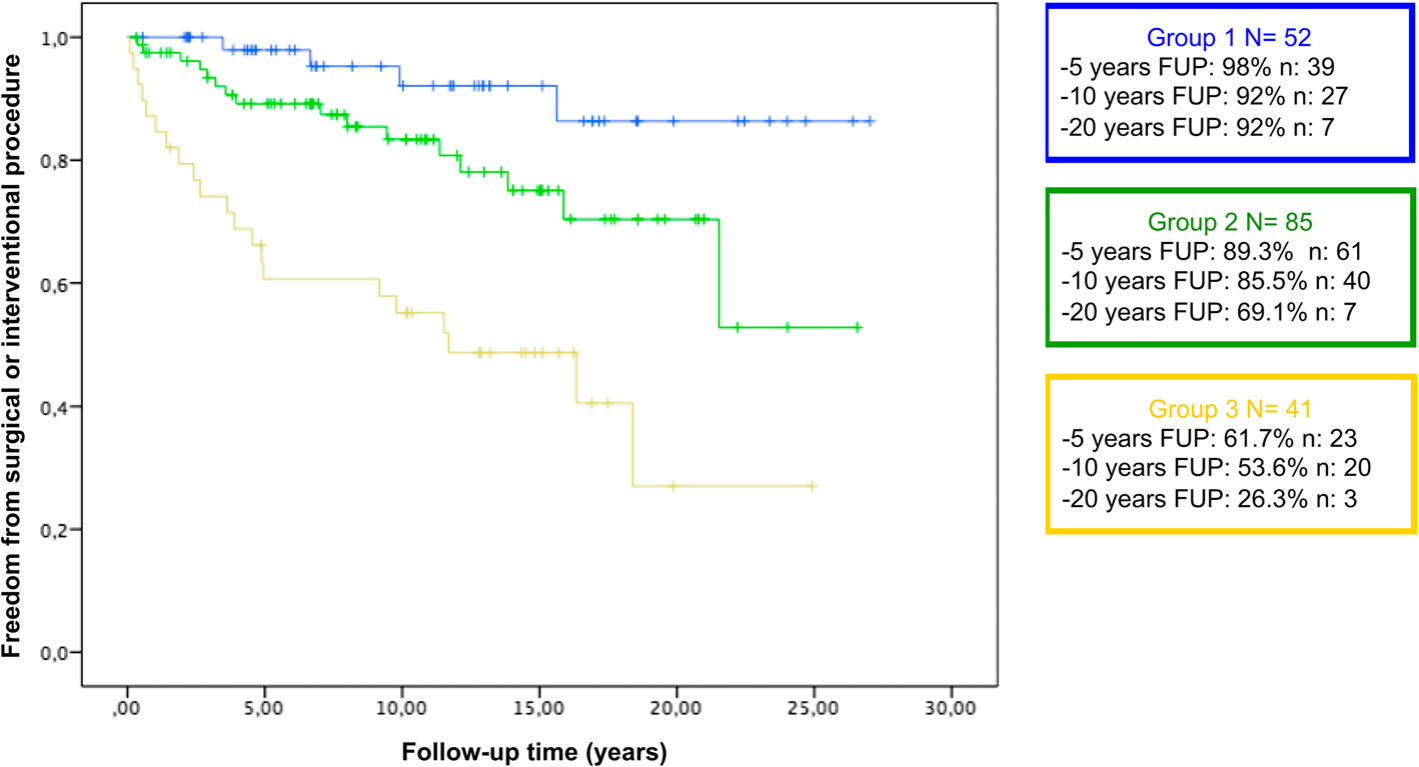
Kaplan–Meier freedom from any surgical or interventional procedure in group 1, 2 and 3

**Fig. 2:**
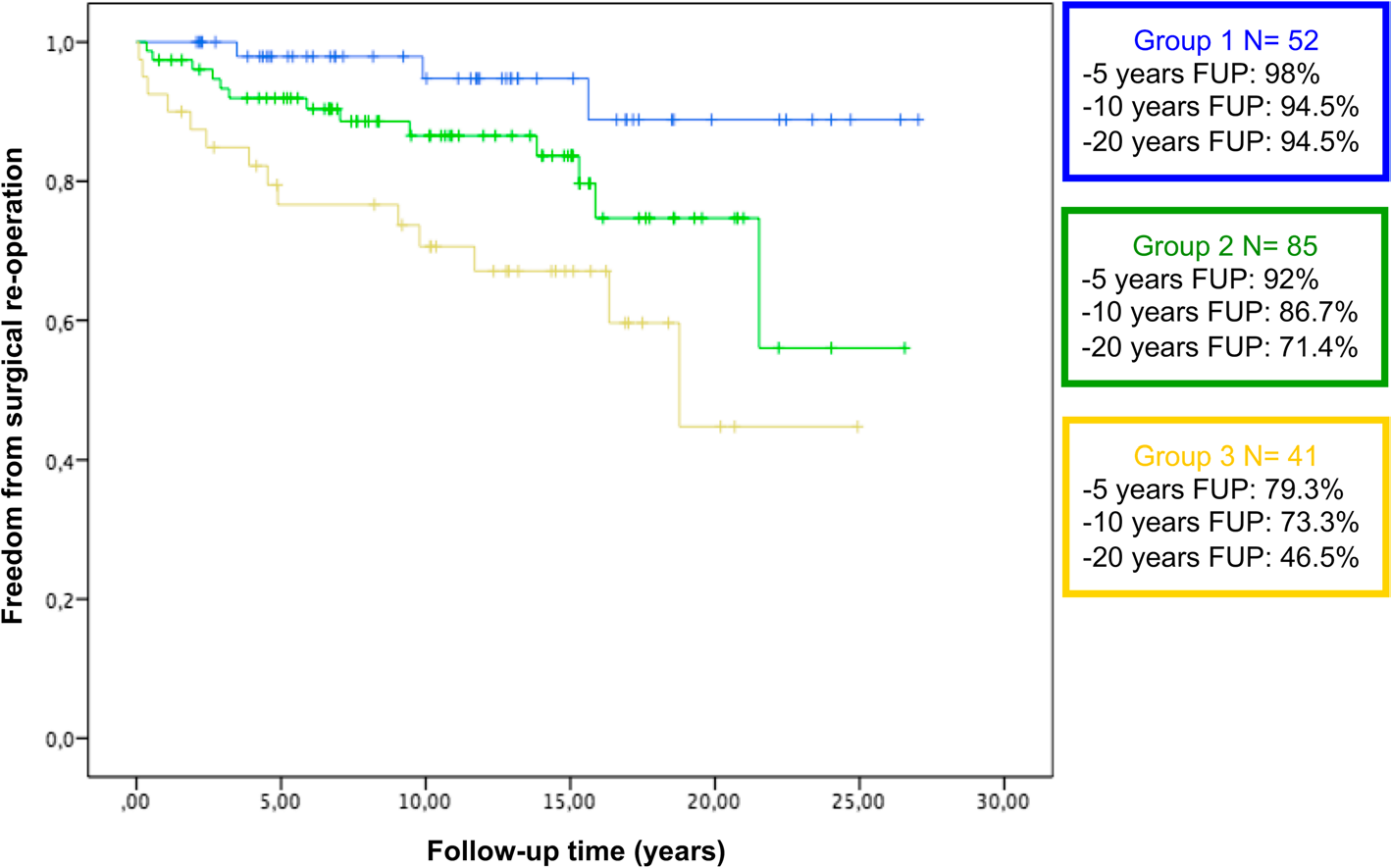
Kaplan–Meier freedom from surgical re-operation in group 1, 2 and 3

**Fig. 3:**
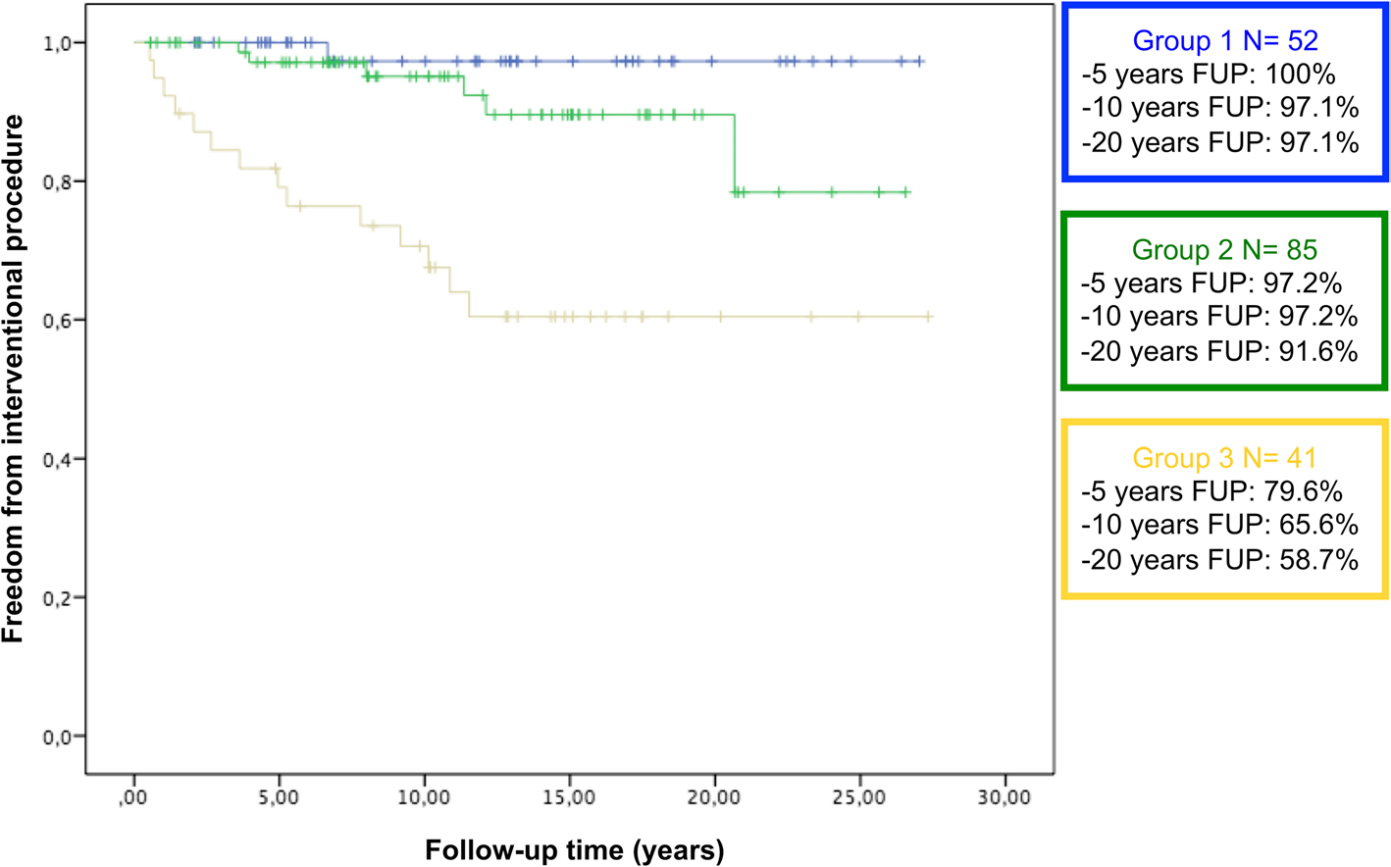
Kaplan–Meier freedom from interventional procedure in group 1, 2 and 3

At Kaplan–Meier analysis the freedom from combined surgical or interventional procedures was significantly different between the 3 groups: Chi-square: 30; *P* < 0.001 (Fig. 1). The freedom from only surgical procedure or only interventional procedure was also significantly different between the groups, respectively Chi-square: 11.8; *P* = 0.003 (Fig. 2), Chi-square: 28.8; *P* < 0.001 (Fig. 3).

The univariate Cox regression analysis identified only PAs and PV hypoplasia as predictors of post-repair requirement of combined surgical + interventional procedures (Table 3), surgical procedure or interventional procedure alone (Tables 3, 4 and 5). At multivariate Cox regression PAs and PV hypoplasia confirmed to be independent predictors for subsequent procedures, respectively HR:3.1, CI: 1.06–9.1, *p* = 0.03 and HR:3.13, CI: 1.66–5.96, *p* < 0.001. Pulmonary valve hypoplasia was associated with the need of further surgical procedures (HR:3.4, CI: 0.993–11.7, *p* = 0.05) and PAs hypoplasia for interventional procedures (HR:5.8, CI: 2.16–15.68, *p* < 0.001).

**Table 3:** Univariate and multivariate Cox analysis for post repair procedures (surgical or interventional)

	**HR**	**95% CI HR**	***P***	**HR**	**95% CI HR**	***P***
**Gender (male)**	0.82	0.437 – 1.543	0.54			
**Previous palliation**	0.74	0.343–1.609	0.45			
**Age at primary repair**	0.85	0.360 – 2.003	0.73			
**Type of primary repair**	1.859	0.885 – 3.905	0.1			
**- TAP**						
**- Infundibular patch or commisurotomy**						
**PV annulus hypoplasia**	4.57	1.626 – 12.881	**0.004**	2.85	0.972–8,27	**0.05**
**PAs hypoplasia**	4.45	2.239 – 8.286	** < 0.001**	3.46	1.819 -6.587	** < 0.001**

*PAs* Pulmonary artery branches, *PV* Pulmonary valve, *TAP* Trans annular patch

**Table 4:** Univariate and multivariate Cox analysis for post-surgical procedures

	**HR**	**95% CI HR**	***P***	**HR**	**95% CI HR**	***P***
**Gender (male)**	0.48	0.213 – 1.095	0.08			
**Previous shunt**	0.45	0.479 – 5.237	0.45			
**Age at primary repair**	0.6	0.205 – 1.759	0.35			
**Type of primary repair**	1.587	0.674 – 3.733	0.27			
**- TAP**						
**- Infundibular patch or commisurotomy**						
**PV annulus hypoplasia**	4.1	1.24 – 13.75	**0.02**	3.40	0.959–11.785	**0.05**
**PAs hypoplasia**	2.43	1.166 – 5.089	**0.018**	1.903	0.868–3.944	0.1

*PAs* Pulmonary artery branches, *PV* Pulmonary valve, *TAP* Trans annular patch

**Table 5:** Univariate and multivariate Cox analysis for post interventional procedures

	**HR**	**95% CI HR**	***P***	**HR**	**95% CI HR**	***P***
**Gender (male)**	0.76	0.317 – 1.852	0.55			
**Previous shunt**	0.43	0.165 – 1.100	0.078			
**Age at primary repair**	1.14	0.375 – 3.550	0.80			
**Type of primary repair**	4.63	1.217 – 67.7	**0.03**	1.200	0.194–7.429	0.84
**- TAP**						
**- Infundibular patch or commisurotomy**						
**PV annulus hypoplasia**	8.4	1.264 – 70.284	**0.02**	3.043	0.236–39.226	0.39
**PAs hypoplasia**	10.04	3.671 – 27.450	** < 0.001**	7.429	2.61–21.277	** < 0.001**

*PAs* Pulmonary artery branches, *PV* Pulmonary valve, *TAP* Trans annular patch

## Discussion

In this retrospective single-center study, we proposed a simple anatomic classification based on the size of the pulmonary valve annulus and pulmonary artery branches. We evaluated its impact on the subsequent requirement of surgical or interventional procedures, and on adverse cardiac events at medium term follow-up.

In the last decades, many efforts have been made with the aim to optimize and standardize the surgical treatment of TOF, in order to improve short-term and long-term results [9, 10]. The ideal TOF repair should be suitable for infants and should provide good relief of the RVOT obstruction, atrial and ventricular septation. It should also avoid an extensive ventriculotomy and last but not least, it should preserve the function of the pulmonary valve. Nowadays, the transatrial or transatrial-transpulmonary approach is utilized in most centers with excellent results. Neonatal repair is feasible with acceptable results, but it more often requires transannular patch, resulting in worse event-free survival [11].

Tetralogy of Fallot palliation in the current era is restricted to a limited group of patients, most of them are neonates or babies with small body weight. In our study, only 16% of the patients required a palliative procedure (BT shunt) before primary repair, and this percentage is compatible with most of the recent experiences from many other centers [12–15]. Also, in conformity with the current literature, our approach of primary repair with TAP during infancy is the most commonly used [16]. Lastly, it is known that the TAP is the preferred procedure mainly in patients with hypoplastic PV annulus [17], and this was indeed also the most frequent repair in our cohort of patients (68%). Repair without ventriculotomy (19% of cases) and repair with infundibular patch (16% of cases) were the other common techniques that we used.

Although it’s well established that TOF presents a wide range of anomalies, the follow-up recommendations, mainly in adult patients, are tailored more on the postoperative results [18] without taking in account the native anatomy. In our population, we tested the impact of a simple anatomical classification on the surgical history and mid-term outcomes of patients who underwent TOF repair. In the setting of pre-operative native anatomy, in group 1 (normal size PV annulus and PAs) only 4 (6%) patients required a TAP. In 2 of them, the annulus was normal in size but the commissurotomy was inadequate due to severe valve dysplasia while in the others 2 patients the annulus was intraoperatively found to be smaller than expected from preoperative echocardiogram. Conversely in group 2 and 3 most of the patients received a TAP, 91% and 94% respectively, with 46 patients (70%) in group 3 requiring also a PAs enlargement due to hypoplastic branches. Patients from group 1 and group 2 almost never required PAs enlargement.

When considering mid-term follow up, the patients in group 1 had a low incidence of surgical re-interventions (5.7%) and only 1 required an interventional procedure. Freedom from any procedure was 98% at 5 years, 92% at 10 years and 92% at 20 years (Fig. 1), with no adverse events. These results show that a favorable anatomy, defined as PV and PAs size with z-score more than -2, leads to very low incidence of reoperations or interventional procedures an no significant adverse events in the following years. Conversely a significant higher incidence of surgical or interventional procedures was found in group 2 and 3. For group 2, freedom from any procedure was 89.3% at 5 years, 85.5% at 10 years and 69.1% at 20 years (Fig. 1). Most of the cases required surgical re-interventions to relieve RVOT restenosis at mid-term follow-up and to readdress PV regurgitation in long term follow-up. Only 2 (2.3%) patients required multiple procedures. In this group, the number of interventional procedures was limited to 5, mainly PAs balloon angioplasties and RVOT stent implantation. In the same group, only 3 patients experienced adverse cardiac events. In group 3 freedom from any procedure was 61.7% at 5 years, 53.6% at 10 years and 26.3% at 20 years. These patients required re-interventions due to PV regurgitation and/or RVOTO and/or stenosis of PAs. Among them, 20% required multiple combined surgical and interventional procedures, and 5 patients (12.2%) underwent 3 or 4 repeated procedures. In terms of adverse cardiac events, only one patient presented signs of heart failure 20 years later.

In the present study, PV annulus hypoplasia was an independent predictor for redo surgical procedures, in accordance with previous studies [19]; while PAs hypoplasia was an independent predictor of subsequent interventional procedures.

### Clinical implications

In this study we propose a simple anatomical classification that could be provided by a routine echocardiogram study focused on the size of the pulmonary valve annulus and pulmonary artery branches. In our population, we found a helpful correlation between this classification and the surgical results and mid-term outcomes. Actually, patients with preserved PV function and without residual stenosis (group 1), no adverse events or subsequent re-interventions occurred after 5 years, and in most of them even after 10 years. PV hypoplasia is the most frequent anatomical feature and surgical treatment inevitably requires a TAP with possible consequent PV regurgitation. In patients with TAP and normal size PAs (group 2), the number of re-interventions within 5 years was low and mainly due to residual RVOT obstruction; patients with PV and PAs hypoplasia (group 3), are the most challenging, as they underwent more frequently surgical and/or interventional procedures after the complete repair.

If these findings would be confirmed by larger prospective multicenter randomized studies, it would be reasonable to tailor both the counseling and the follow-up of patients who undergo TOF repair according to the native anatomy rather than according to a standardized protocol. For instance, in TOF patient with TAP and good size PAs without residual stenosis, even if with so-called “free pulmonary regurgitation”, would require very few cardiology out-patient clinics, at least in the first 5 years post-surgical repair. Because of the quite small sample size and the limited follow-up time we couldn’t assess the impact of the anatomical classification on long-term adverse outcomes. However, previous studies identified multiple cardiac operations as one of the risk factors for ventricular tachycardia and SCD [20] therefore we could speculate that the risk for adverse outcome of patients in the group 3 (with more subsequent operations) could be higher in comparison with the others 2 groups.

### Study limitations

We acknowledge that our study presents the limitations of the retrospective nature and of the relatively low sample size, however the fact all patients were operated in the same center certainly reduces surgical strategy bias. Patients lost at follow-up were older compared with the remaining population, therefore the long-term results should be interpreted cautiously. Larger longitudinal studies are warranted to better investigate the impact of the native anatomy on adverse outcomes at long-term follow-up in this challenging population.

## Conclusion

Tetralogy of Fallot entails a wide spectrum of native anatomy variations. These affect the surgical strategy and the subsequent follow-up, in particular the incidence of adverse cardiac events and timing/number of subsequent surgical/interventional procedures. Therefore, if our data will be confirmed by larger prospective studies, the counseling and the appropriate follow-up at least at medium-term follow-up might be tailored according to the native anatomy rather than according to a standardized protocol.

## Supplementary Information

**Additional file 1: Supplementary table 1.** Comparison between patients lost at follow-up and patients with complete follow-up.

## Data Availability

The data that support the findings of this study are available from the corresponding author upon reasonable request.

## Abbreviations

TOF: Tetralogy of Fallot
VSD: Ventricular septal defect
RVOT: Right ventricular outflow tract
PV: Pulmonary valve
PAs: Pulmonary artery branches
HF: Heart failure
TTE: Transthoracic echocardiogram
VT/NSVT: Sustained and non-sustained ventricular tachycardia
ICD: Implantable cardioverter-defibrillator
CPET: Cardio-pulmonary exercise test
BT: Blalock-Taussig shunt
PTFE: Polytetrafluoroethylene
TAP: Transannular patch
DORV: Double outlet right ventricle
PDA: Patent ductus arteriosus
IQR: Interquartile range
