# Supplementary material for: The impact of native Fallot anatomy on future therapeutic requirements and outcomes at follow-up
Source: Cardiovasc Ultrasound. 2021 Jun 19;19:23. doi: 10.1186/s12947-021-00249-y (PMC8214257; doi:10.1186/s12947-021-00249-y)
Supplement: Supplementary file 1 — Additional file 1: Supplementary table 1. Comparison between patients lost at follow-up and patients with complete follow-up. [file 12947_2021_249_MOESM1_ESM.doc]

Supplementary table 1: Comparison between patients lost at follow-up and patients with complete follow-up.

|  | **Follow-up**  **(178)** | **No follow-up**  **(61)** | ***p*** |
| --- | --- | --- | --- |
| **Gender male (%)** | 105 (59) | 43 (69.4) | 0.09 |
| **Diagnosis**  **TOF (%)**  **DORV-Fallot type (%)** | 170 (95.5)  8 (4.5) | 57 (91.9)  5 (8.1) | 0.2 |
| **Previous BT shunt (%)** | 28 (15,7) | 10 (16.1) | 0.8 |
| **Type of primary repair**  **TAP (%)**  **Infundibular patch/ PV commissurotomy (%)** | 119 (66.9)  17 (27.9) | 44 (72.1)  59 (33.1) |  |

DORV: double outlet right ventricle, PV: pulmonary valve, TAP: transannular patch, TOF: tetralogy of Fallot.
